# Supplementary material for: A mosaic adeno-associated virus vector as a versatile tool that exhibits high levels of transgene expression and neuron specificity in primate brain
Source: Nat Commun. 2023 Aug 8;14:4762. doi: 10.1038/s41467-023-40436-1 (PMC10409865; doi:10.1038/s41467-023-40436-1)
Supplement: Supplementary file 1 — Supplementary Information [file 41467_2023_40436_MOESM1_ESM.pdf]

Supplementary Information

A mosaic adeno-associated virus vector as a versatile tool that exhibits high levels of transgene expression and neuron specificity in primate brain

Kei Kimura[1], Yuji Nagai[2], Gaku Hatanaka[3,4], Yang Fang[3,4], Soshi Tanabe[1], Andi Zheng[1], Maki Fujiwara[1], Mayuko Nakano[1], Yukiko Hori[2], Ryosuke F. Takeuchi[3,4], Mikio Inagaki[3,4], Takafumi Minamimoto[2], Ichiro Fujita[3,4], Ken-ichi Inoue[1,5*], Masahiko Takada[1*]

[1]Systems Neuroscience Section, Department of Neuroscience, Primate Research Institute, and Center for the Evolutionary Origins of Human Behavior, Kyoto University, Inuyama, Aichi 484-8506, Japan

[2]Department of Functional Brain Imaging, National Institutes for Quantum Science and Technology, Chiba 263-8555, Japan

[3]Laboratory for Cognitive Neuroscience, Graduate School of Frontier Biosciences, Osaka University, 1-4 Yamadaoka, Suita, Osaka 565-0871, Japan

[4]Center for Information and Neural Networks, National Institute of Information and Communications Technology and Osaka University, 1-4 Yamadaoka, Suita, Osaka 565-0871, Japan

[5]PRESTO, Japan Science and Technology Agency, Kawaguchi, Saitama 332-0012, Japan

*Co-corresponding authors: Ken-ichi Inoue, inoue.kenichi.6z@kyoto-u.ac.jp

Masahiko Takada, takada.masahiko.7x@kyoto-u.ac.jp

CONTENT:

Supplementary figures 1 - 3

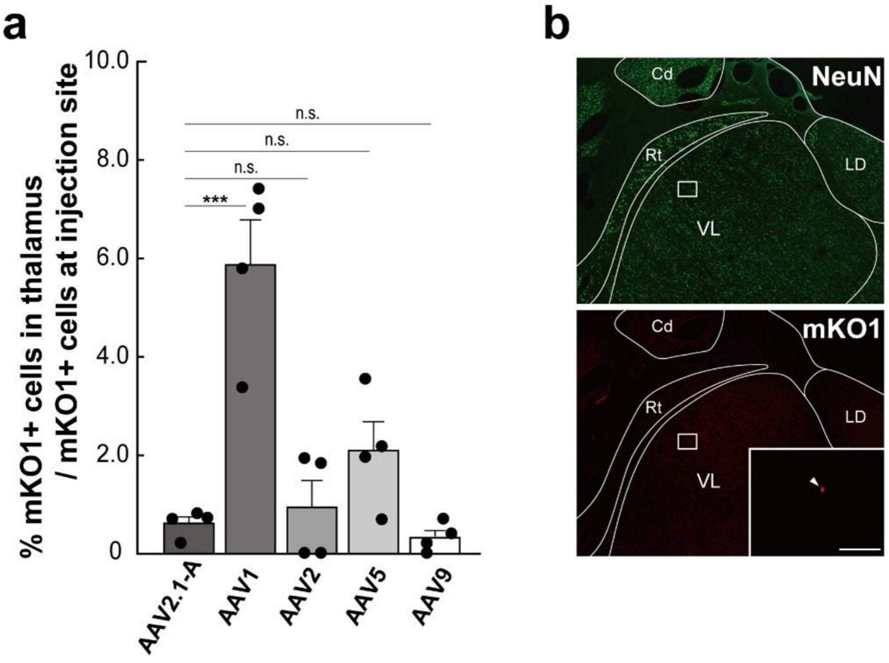

Supplementary Figure 1. Retrograde or anterograde transneuronal labeling of thalamic neurons after cortical injections of AAV vectors.

(**a**) Comparison of the extent of retrograde or anterograde transneuronal labeling of thalamic neurons in the rat after cortical injections of the AAV2.1-A, AAV1, AAV2, AAV5, and AAV9 vectors. Data are shown as the ratio of labeled neurons in the thalamus (especially in the ventrolateral nucleus) to those at the injection site. Expressed as the mean for four rats. Each dot represents the ratio for each individual. (**b**) Double fluorescence histochemistry for NeuN (upper) and mKO1 (lower) in a coronal section through the ventrolateral nucleus (VL) of the monkey thalamus after cortical injection of the AAV2.1-A vector. Shown are an immunofluorescence image of NeuN labeling (green) and a native-fluorescence image of mKO1 labeling (red) taken from Monkey C. Only very rarely is found cell body labeling as specified by an arrowhead in an inset (zoom-up of the rectangle in the original images). Cd, caudate nucleus; LD, laterodorsal nucleus of the thalamus; Rt, reticular nucleus of the thalamus. Scale bar, 100 μm. Significant

difference from the AAV2.1 vector (one-way ANOVA followed by post hoc Dunnett's test): ***$P < 0.001$, n.s. $P > 0.05$. Source data are provided with this paper. The exact P values also are available as a Source Data file. Error bars, SEM. n=4 biologically independent animals.

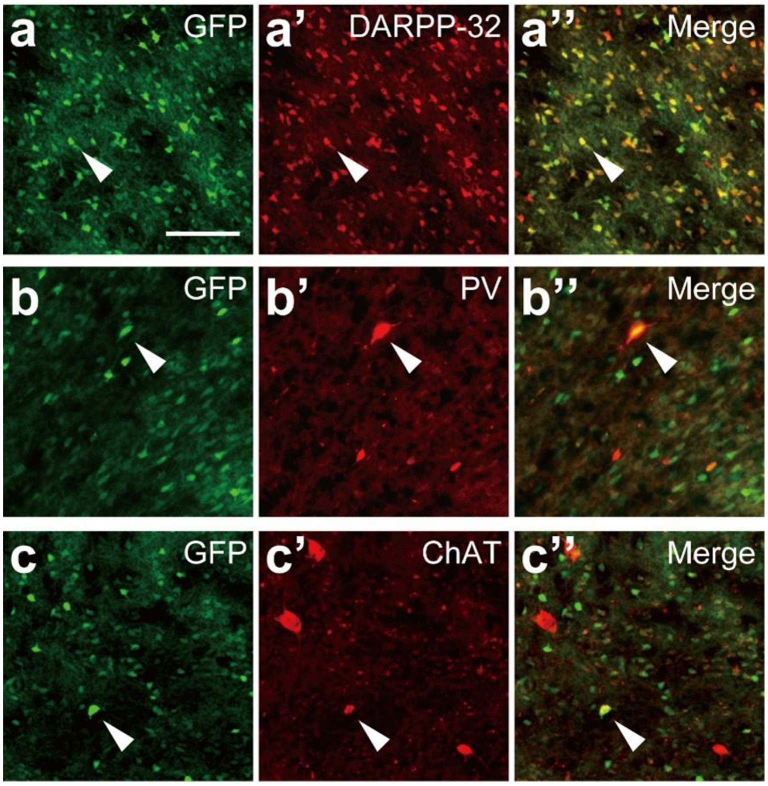

Supplementary Figure 2. Cell types of striatal neurons transduced with AAV2.1-A vector.

Double fluorescence histochemistry for GFP native-fluorescence (green; a-c) and immunofluorescence for DARPP-32, PV, or ChAT (red; a'-c') at the site of AAV2.1-A vector injection in the striatum. Data were obtained in Monkey E. Shown in yellow (Merge; a"-c") are double-labeled neurons which are pointed to with arrowheads. Scale bar, 100 µm.

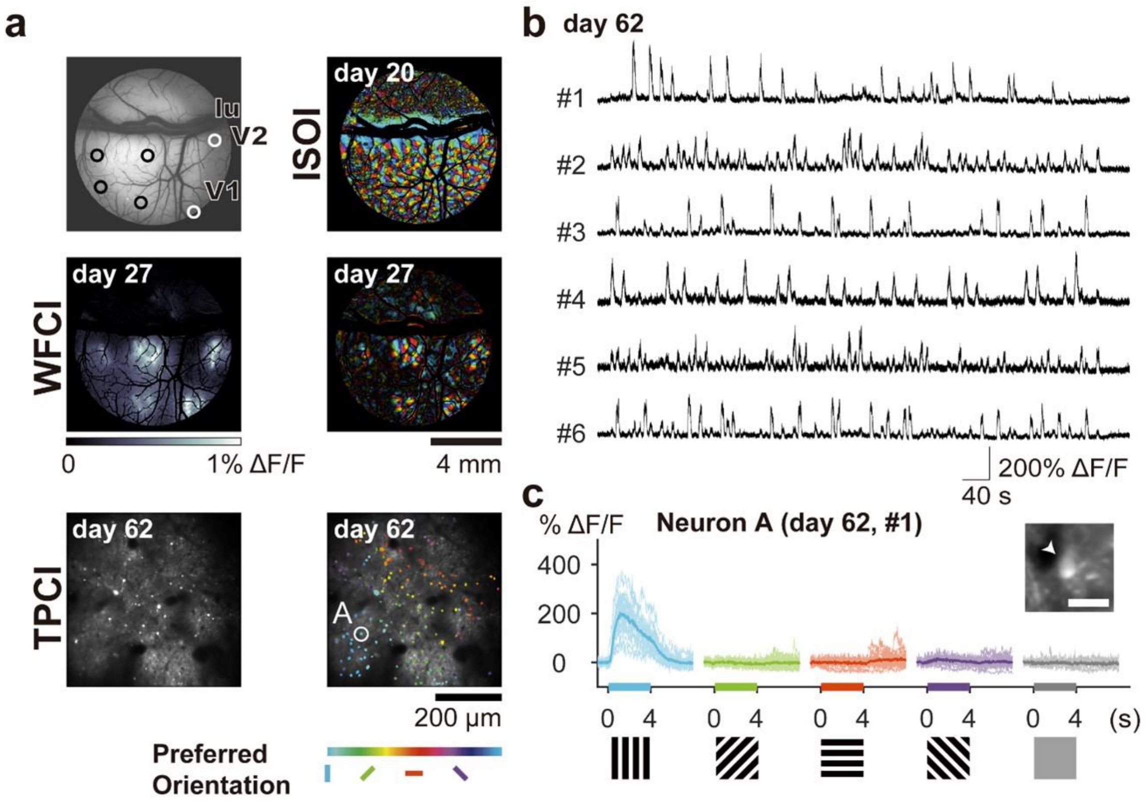

Supplementary Figure 3. *In vivo* calcium imaging of visual cortical neuron activity for determining optimal conditions.

(**a**) Upper row: Left, six loci of GCaMP6s-expressing AAV2.1-A vector injections (denoted by open circles; AAV2.1-A-CaMKIIα-GCaMP6s in black, AAV2.1-A-Syn-GCaMP6s in white) in Monkey G. lu, lunate sulcus; Right, intrinsic signal optical imaging (ISOI) for visualizing an orientation map of the visual cortex at 20 days post-injection. Middle row: One-photon wide-field calcium imaging (WFCI). Left, vector injection sites at 27 days post-injection visualized by selecting the maximum fluorescence changes ($\Delta F/F$) for each pixel across all stimulus conditions; Right, orientation map visualized at 27 days post-injection. The color code indicates the preferred orientation determined for each pixel. Note that this map is basically consistent with the one obtained from ISOI (see the upper right). Lower row: Two-photon calcium imaging (TPCI). Left, structural image

of the recording sites at 62 days post-injection obtained by averaging the fluorescence across all frames for a recording session. Right, orientation map at the single-neuron resolution at 62 days post-injection. The color code represents the preferred orientation determined for each neuron. (**b**) Time-dependent changes ($\Delta F/F$) in fluorescent signals obtained from six exemplified neurons in the V1 recorded at 62 days after the vector injection. These calcium transients were recorded in response to visual stimuli. (**c**) Responses of fluorescent signals of a V1 neuron (Neuron A) to drifting gratings of different orientations. The responses to the same orientation with opposite moving directions are superimposed. (Inset) Arrowhead points to the imaged neuron. Data were obtained in Monkey G. Scale bar, 20 μm.
