# Supplementary material for: A mosaic adeno-associated virus vector as a versatile tool that exhibits high levels of transgene expression and neuron specificity in primate brain
Source: Nat Commun. 2023 Aug 8;14:4762. doi: 10.1038/s41467-023-40436-1 (PMC10409865; doi:10.1038/s41467-023-40436-1)
Supplement: Supplementary file 3 — Description of Additional Supplementary Files [file 41467_2023_40436_MOESM3_ESM.pdf]

Description of Additional Supplementary Files

File name: Supplementary Data 1

Description: Regents and antibodies used in this study.
