# Supplementary material for: A mosaic adeno-associated virus vector as a versatile tool that exhibits high levels of transgene expression and neuron specificity in primate brain
Source: Nat Commun. 2023 Aug 8;14:4762. doi: 10.1038/s41467-023-40436-1 (PMC10409865; doi:10.1038/s41467-023-40436-1)
Supplement: Supplementary file 5 — Reporting Summary [file 41467_2023_40436_MOESM5_ESM.pdf]

nature portfolio

Corresponding author(s): Ken-ichi Inoue, Masahiko Takada

Last updated by author(s): Jun 16, 2023

Reporting Summary

Nature Portfolio wishes to improve the reproducibility of the work that we publish. This form provides structure for consistency and transparency in reporting. For further information on Nature Portfolio policies, see our Editorial Policies and the Editorial Policy Checklist.

Statistics

For all statistical analyses, confirm that the following items are present in the figure legend, table legend, main text, or Methods section.

n/a	Confirmed	
☐	☒	The exact sample size (n) for each experimental group/condition, given as a discrete number and unit of measurement
☐	☒	A statement on whether measurements were taken from distinct samples or whether the same sample was measured repeatedly
☐	☒	The statistical test(s) used AND whether they are one- or two-sided<br>*Only common tests should be described solely by name; describe more complex techniques in the Methods section.*
☐	☒	A description of all covariates tested
☐	☒	A description of any assumptions or corrections, such as tests of normality and adjustment for multiple comparisons
☐	☒	A full description of the statistical parameters including central tendency (e.g. means) or other basic estimates (e.g. regression coefficient) AND variation (e.g. standard deviation) or associated estimates of uncertainty (e.g. confidence intervals)
☐	☒	For null hypothesis testing, the test statistic (e.g. F, t, r) with confidence intervals, effect sizes, degrees of freedom and P value noted<br>*Give P values as exact values whenever suitable.*
☒	☐	For Bayesian analysis, information on the choice of priors and Markov chain Monte Carlo settings
☒	☐	For hierarchical and complex designs, identification of the appropriate level for tests and full reporting of outcomes
☒	☐	Estimates of effect sizes (e.g. Cohen's d, Pearson's r), indicating how they were calculated

Our web collection on statistics for biologists contains articles on many of the points above.

Software and code

Policy information about availability of computer code

Data collection	Data for the experiment of intensity analysis was collected using custom Matlab code (Mathworks, Matlab2021a).<br>The custom code is available as the supplemental file in the manuscript.
Data analysis	We used Stereo Investigator software (MBF Biosciences), Neurolucida software (MBF Biosciences) for data analysis.

For manuscripts utilizing custom algorithms or software that are central to the research but not yet described in published literature, software must be made available to editors and reviewers. We strongly encourage code deposition in a community repository (e.g. GitHub). See the Nature Portfolio guidelines for submitting code & software for further information.

Data

Policy information about availability of data

All manuscripts must include a data availability statement. This statement should provide the following information, where applicable:

- Accession codes, unique identifiers, or web links for publicly available datasets
- A description of any restrictions on data availability
- For clinical datasets or third party data, please ensure that the statement adheres to our policy

The source data generated in this study is available as the Source Data file attached with the manuscript.

Human research participants

Policy information about studies involving human research participants and Sex and Gender in Research.

Reporting on sex and gender	N/A
Population characteristics	N/A
Recruitment	N/A
Ethics oversight	N/A

Note that full information on the approval of the study protocol must also be provided in the manuscript.

Field-specific reporting

Please select the one below that is the best fit for your research. If you are not sure, read the appropriate sections before making your selection.

☒ Life sciences ☐ Behavioural & social sciences ☐ Ecological, evolutionary & environmental sciences

For a reference copy of the document with all sections, see nature.com/documents/nr-reporting-summary-flat.pdf

Life sciences study design

All studies must disclose on these points even when the disclosure is negative.

Sample size	In the experiments in monkeys, we determined the sample size for the each series of our experiments as two macaque monkeys. We determined the sample size based on lots of typical primate neuroscience researches. We used eight macaque monkeys because we had four different series of experiments. We do not use any statistical methods to predetermine sample size. In the experiments in rats, the number of animal used was four. To use of statistical analysis (dunnett's t test) among multiple groups, we need to prepare four individuals for one group at least.
Data exclusions	No data were excluded from the analyses.
Replication	All attempts at replication were successful. Results were consistent with previous studies and among two or four monkeys for each experiment in the current study.
Randomization	No experimental groupings were used for this study.
Blinding	No applicable because no grouping was performed.

Behavioural & social sciences study design

All studies must disclose on these points even when the disclosure is negative.

Study description	N/A
Research sample	N/A
Sampling strategy	N/A
Data collection	N/A
Timing	N/A
Data exclusions	N/A
Non-participation	N/A
Randomization	N/A

Ecological, evolutionary & environmental sciences study design

All studies must disclose on these points even when the disclosure is negative.

Study description	N/A
Research sample	N/A
Sampling strategy	N/A
Data collection	N/A
Timing and spatial scale	N/A
Data exclusions	N/A
Reproducibility	N/A
Randomization	N/A
Blinding	N/A

Did the study involve field work? ☐ Yes ☒ No

Reporting for specific materials, systems and methods

We require information from authors about some types of materials, experimental systems and methods used in many studies. Here, indicate whether each material, system or method listed is relevant to your study. If you are not sure if a list item applies to your research, read the appropriate section before selecting a response.

Materials & experimental systems

n/a	Involved in the study	
☐	☒	Antibodies
☐	☒	Eukaryotic cell lines
☒	☐	Palaeontology and archaeology
☐	☒	Animals and other organisms
☒	☐	Clinical data
☒	☐	Dual use research of concern

Methods

n/a	Involved in the study	
☒	☐	ChIP-seq
☒	☐	Flow cytometry
☒	☐	MRI-based neuroimaging

Antibodies

Antibodies used	Guinea pig polyclonal anti-NeuN (Millipore, Cat# ABN90); Mouse monoclonal anti-S100β (Sigma-Aldrich, Cat# s2532); Donkey-anti-GP DyLight405 (Jackson ImmunoResearch Laboratories, Inc., Cat# 706-475-148); Donkey-anti-Ms AF647 (Jackson ImmunoResearch Laboratories, Inc., Cat# 706-605-148); Chicken monoclonal anti-GFP (Abcam, Cat# ab13970); Rabbit monoclonal anti-DARPP-32 (Cell Signaling Technology, Cat# 2302); Mouse monoclonal anti-PV (Sigma-Aldrich, Cat# P3088); Goat monoclonal anti-ChAT (Sigma-Aldrich, Cat# AB144P); Rabbit monoclonal anti-GFP (Invitrogen, Cat# G10362); Rabbit polyclonal anti-M3 (Atlas Antibodies, Cat# HPA024106); Donkey-anti-Rb AF555 (Invitrogen, Cat# A31572); Donkey-anti-Ck AF488 (Jackson ImmunoResearch Laboratories, Inc., Cat# 703-545-155); Donkey-anti-Ms AF555 (Invitrogen, Cat# A31570); Rabbit polyclonal anti-c-fos (Abcam, Cat# ab190289); Donkey-anti-Rb Biotin (Jackson ImmunoResearch Laboratories, Inc., Cat# 711-065-152)
Validation	The anti-c-fos primary antibody was applied with primate brain sections by Alexander, L. et al (Neuron, 2019).<br>The other commercial antibodies were validated by manufacturer, as follows:<br>Guinea pig polyclonal anti-NeuN: https://www.merckmillipore.com/JP/ja/product/Anti-NeuN-Antibody,MM_NF-ABN90<br>Mouse monoclonal anti-S100β: https://www.sigmaaldrich.com/JP/en/product/sigma/s2532<br>Donkey-anti-GP DyLight405: https://www.jacksonimmuno.com/catalog/products/706-475-148<br>Donkey-anti-Ms AF647: https://www.jacksonimmuno.com/catalog/products/706-605-148<br>Chicken monoclonal anti-GFP: https://www.abcam.com/products/primary-antibodies/gfp-antibody-ab13970.html<br>Rabbit monoclonal anti-DARPP-32: https://www.cellsignal.com/products/primary-antibodies/darpp-32-antibody/2302<br>Mouse monoclonal anti-PV: https://www.sigmaaldrich.com/JP/en/product/sigma/p3088<br>Goat monoclonal anti-ChAT: https://www.sigmaaldrich.com/JP/en/product/mm/ab144p<br>Rabbit monoclonal anti-GFP: https://www.thermofisher.com/antibody/product/GFP-Antibody-Recombinant-Monoclonal/G10362<br>Rabbit polyclonal anti-M3: https://www.atlasantibodies.com/products/antibodies/primary-antibodies/triple-a-polyclonals/chrm3-

antibody-hpa024106/
Donkey-anti-Rb AF555: https://www.thermofisher.com/antibody/product/Donkey-anti-Rabbit-IgG-H-L-Highly-Cross-Adsorbed-Secondary-Antibody-Polyclonal/A-31572
Donkey-anti-Ck AF488: https://www.jacksonimmuno.com/catalog/products/703-545-155
Donkey-anti-Ms AF555: https://www.thermofisher.com/antibody/product/Donkey-anti-Mouse-IgG-H-L-Highly-Cross-Adsorbed-Secondary-Antibody-Polyclonal/A-31570
Rabbit polyclonal anti-c-fos: https://www.abcam.com/products/primary-antibodies/c-fos-antibody-bsa-free-ab190289.html
Donkey-anti-Rb Biotin: https://www.jacksonimmuno.com/catalog/products/711-065-152

Eukaryotic cell lines

Policy information about cell lines and Sex and Gender in Research

Cell line source(s)	AAV-293 cells were purchased from Stratagene.
Authentication	AAV-293 cells were authenticated by the supplier (Stratagene) using morphology and growth characteristics.
Mycoplasma contamination	AAV-293 cells have been tested and shown to be free from mycoplasma.
Commonly misidentified lines (See ICLAC register)	No commonly misidentified cell lines were used.

Palaeontology and Archaeology

Specimen provenance	N/A
Specimen deposition	N/A
Dating methods	N/A

☐ Tick this box to confirm that the raw and calibrated dates are available in the paper or in Supplementary Information.

Ethics oversight	N/A

Note that full information on the approval of the study protocol must also be provided in the manuscript.

Animals and other research organisms

Policy information about studies involving animals; ARRIVE guidelines recommended for reporting animal research, and Sex and Gender in Research

Laboratory animals	Rhesus monkey (Macaca mulatta), two female, 8 and 18 years old, 5.6-7.8 kg; Cynomolgus monkey (Macaca fascicularis), one male, 4 years old,5.2 kg; Japanese monkeys (Macaca fuscata), one male, four females, 3-12 years old, 5.3-8.3 kg, Wister rat, twenty males, 12-13 weeks old, 260-318g
Wild animals	No wild animals were used in this study.
Reporting on sex	All animals sex were reported above.
Field-collected samples	No field-collected samples were used in this study.
Ethics oversight	For the first set of our experiments with Monkeys A-D and the rats on gene transduction properties of mosaic AAV vectors, the experimental protocol was approved by the Animal Welfare and Animal Care Committee of the Primate Research Institute, Kyoto University (Permission Number: 2018-046), and all experiments were conducted according to the Guidelines for Care and Use of Nonhuman Primates established by the Primate Research Institute, Kyoto University (2010). For the second set of our experiments with Monkeys E and F on application of AAV2.1-A vector to chemogenetic manipulation, the experimental protocol was approved by the Animal Ethics Committee of the National Institutes for Quantum Science and Technology (Permission Number: 11-1038-11), and all experiments were conducted in accordance with the Guide for the Care and Use of Nonhuman Primates in Neuroscience Research (Japan Neuroscience Society; https://www.jnss.org/en/animal_primates). For the third set of our experiments with Monkeys G and H on application of AAV2.1-A vector to in vivo calcium imaging, the experimental protocol was approved by the Animal Experiment Committee of Osaka University (Permission Number: FBS-18-005), and all experiments were conducted according to the Guidelines for Animal Experiments established by Osaka University.

Note that full information on the approval of the study protocol must also be provided in the manuscript.

Clinical data

Policy information about clinical studies

All manuscripts should comply with the ICMJE guidelines for publication of clinical research and a completed CONSORT checklist must be included with all submissions.

Clinical trial registration	N/A
Study protocol	N/A
Data collection	N/A
Outcomes	N/A

Dual use research of concern

Policy information about dual use research of concern

Hazards

Could the accidental, deliberate or reckless misuse of agents or technologies generated in the work, or the application of information presented in the manuscript, pose a threat to:

No	Yes	
☒	☐	Public health
☒	☐	National security
☒	☐	Crops and/or livestock
☒	☐	Ecosystems
☒	☐	Any other significant area

Experiments of concern

Does the work involve any of these experiments of concern:

No	Yes	
☒	☐	Demonstrate how to render a vaccine ineffective
☒	☐	Confer resistance to therapeutically useful antibiotics or antiviral agents
☒	☐	Enhance the virulence of a pathogen or render a nonpathogen virulent
☒	☐	Increase transmissibility of a pathogen
☒	☐	Alter the host range of a pathogen
☒	☐	Enable evasion of diagnostic/detection modalities
☒	☐	Enable the weaponization of a biological agent or toxin
☒	☐	Any other potentially harmful combination of experiments and agents

ChIP-seq

Data deposition

☐ Confirm that both raw and final processed data have been deposited in a public database such as GEO.

☐ Confirm that you have deposited or provided access to graph files (e.g. BED files) for the called peaks.

Data access links<br>*May remain private before publication.*	N/A
Files in database submission	N/A
Genome browser session<br>(e.g. UCSC)	N/A

Methodology

Replicates	N/A
Sequencing depth	N/A

Antibodies	N/A
Peak calling parameters	N/A
Data quality	N/A
Software	N/A

Magnetic resonance imaging

Experimental design

Design type	N/A
Design specifications	N/A
Behavioral performance measures	N/A

Acquisition

Imaging type(s)	N/A
Field strength	N/A
Sequence & imaging parameters	N/A
Area of acquisition	N/A

Diffusion MRI ☐ Used ☒ Not used

Preprocessing

Preprocessing software	N/A
Normalization	N/A
Normalization template	N/A
Noise and artifact removal	N/A
Volume censoring	N/A

Statistical modeling & inference

Model type and settings	N/A
Effect(s) tested	N/A

Specify type of analysis: ☐ Whole brain ☐ ROI-based ☐ Both

Statistic type for inference (See Eklund et al. 2016)	N/A
Correction	N/A

Models & analysis

n/a	Involved in the study
☒	☐ Functional and/or effective connectivity
☒	☐ Graph analysis
☒	☐ Multivariate modeling or predictive analysis
